# Integrating screening for non-communicable diseases and their risk factors in routine tuberculosis care in Delhi, India: A mixed-methods study

**DOI:** 10.1371/journal.pone.0202256

**Published:** 2018-08-23

**Authors:** Tanu Anand, Jugal Kishore, Petros Isaakidis, Himanshu A. Gupte, Gurmeet Kaur, Sneha Kumari, Diwakar Jha, Shekhar Grover

**Affiliations:** 1 Department of Community Medicine, North Delhi Municipal Corporation Medical College, Hindu Rao Hospital, Delhi, India; 2 Department of Community Medicine, Vardhaman Mahavir Medical College & Safdurjung Hospital, Delhi, India; 3 Médecins Sans Frontières, Operational Research Unit, Luxembourg City, Luxembourg; 4 Narotam Sekhsaria Foundation, Mumbai, India; 5 National Institute of Cancer Prevention and Research, Noida, India; Department of Internal Medicine, Federal Teaching Hospital Abakaliki, Ebonyi State, Nigeria, NIGERIA

## Abstract

**Background:**

Evidence supports the integration of prevention and management for tuberculosis (TB) with non-communicable diseases (NCDs). Bi-directional screening for TB and diabetes mellitus (DM) is already implemented in India, a country with a dual burden of TB and NCDs. However, very limited programmatic data are available on the feasibility of adding other NCDs and their risk factors in such screening programme.

**Objective:**

To assess the yield, feasibility, and acceptability of a two-stage integrated screening for NCDs and risk factors for NCDs among patients with TB ≥20 years and treated in DOTS centres of two medical colleges in Delhi, between October 2016 and March 2017.

**Methods:**

It was a mixed-methods, triangulation study with a quantitative component (cross-sectional study using questionnaires, anthropometric measurements and records review) and a qualitative component (descriptive study using interview data).

**Results:**

Amongst 403 patients screened, the prevalence of hypertension was 7% (n = 28) with 20 new cases detected and 8% for DM (n = 32) with 6 new cases diagnosed. The number needed to screen to find a new case was 20 and 63 for hypertension and DM respectively. The most frequent NCD-risk factors were inadequate vegetable (80%) and fruits (72%) intake, alcohol use (34%), use of smokeless tobacco (33%) and smoking (32%). Clustering of four or more risk factors was associated with increasing age and male sex (p<0.05). Both patients and health providers considered the screening relevant and acceptable. However, waiting time and costs involved in blood tests were considered as bothersome by the patients, while health providers perceived increased workload, inadequate medical supplies and inadequate skills and knowledge as key challenges in implementation of the screening.

**Conclusion:**

Integrating screening for NCDs and their risk factors in the existing TB programme produces high yield and it is feasible and acceptable by patients and health providers provided the challenges are overcome.

## Introduction

The global dual burden of tuberculosis (TB) and non-communicable diseases (NCDs) manifests through vulnerability of those with NCDs to TB and through negative effects of NCDs on treatment outcomes for TB.[1] Recent evidence shows links between TB and a number of NCDs such as diabetes mellitus (DM), cardiovascular diseases (CVD), lung cancer and chronic respiratory diseases.[2,3] Further, TB and most NCDs share many risk factors such as smoking, poor diet and harmful use of alcohol, that need to be addressed for effective prevention.[1]

The Global action plan for prevention and control of NCDs 2013–2020, has recognized strong interaction between NCDs and infectious diseases including TB and the consequential need to explore common platforms and approaches to maximize the detection and treatment of co-morbidities.[1,4] Similarly, the need for an integrated, patient-centered care, support and prevention is also key a component of the WHO End TB Strategy.[5] Finally, the 2030 Agenda for Sustainable Development and Universal Health Coverage (UHC) also provides a collaborative framework for integration of NCDs into primary health care.[6]

The burden of NCDs is rising disproportionately in lower and middle income countries (LMIC) and populations.[7] NCD related deaths have increased the most in South-East Asia Region (SEAR) from 6.7 million in 2000 to 8.5 million in 2012.[7] Amongst the SEAR countries, India and Indonesia together account for 80% of NCDs deaths with India contributing to two thirds of this share.[7] Every year roughly 5.8 million Indians die from NCDs.[7]

Additionally, India carries the highest burden of Tuberculosis (TB) in the world. It accounts for more than a quarter of world’s incident cases and deaths for TB.[8] In 2016, 0.4 million deaths out of 1.3 million global deaths due to TB occurred in India.[9] It is evident that India is facing a large burden of NCDs and TB and this presents a strong case for the country to accelerate its efforts in addressing this dual burden in a coordinated and integrated manner.

Bi-directional screening for TB and DM is already implemented in India under the Revised National Tuberculosis Control Programme (RNTCP) and the National Programme for Prevention and Control of Cancer, Diabetes, Cardiovascular Diseases and Stroke (NPCDCS).[9] NPCDCS also endorses opportunistic screening for other NCDs and risk factors for NCDs at any health facility in the country. [10] There is some evidence which suggests that screening of TB patients for DM is feasible at the peripheral level.[11–14] However, to date, very limited programmatic data is available on the implementation and feasibility of adding other NCDs and their risk factors in such screening programme at the level of DOTS centre.[2] Further, it is required to explore the acceptability and relevance of such integrated services both by the providers and patients’ perspective. Synthesis of this evidence will require a combination of both quantitative and qualitative methods.[15]

With this background, we aimed to assess the feasibility, yield and acceptability of a two-stage integrated screening of NCDs and risk factors for NCDs among patients with TB aged ≥20 years and treated in DOTS centres of two medical colleges in Delhi, between October 2016 and March 2017.

## Material and methods

### Study design

It was a mixed methods, triangulation study comprising of a quantitative component (cross-sectional study using questionnaires, anthropometric measurements and records review) and a qualitative component (descriptive study using interview data).

### General setting

The study was conducted in the National Capital Territory (NCT) of Delhi, India. Delhi has 11 revenue districts. Healthcare delivery system in Delhi is complex with multiple agencies providing health services in defined subset of populations in different areas. According to RNTCP Annual Report 2017, there were 55,657 patients registered for TB treatment out of the total population of 17 million during 2016.[16] Most of the NCDs too are prevalent in the city with prevalence of hypertension amongst men and women being 4.2% and 7.6% respectively.[17] Blood sugar level >140 mg/dl have been reported amongst 14.3% and 11.6% men and women respectively [17]

### Study sites

The study sites were two DOTS centres attached to two medical colleges in Delhi. While one DOTS centre was located in North District of Delhi catering to a population of 0.15 million, the other DOTS centre was in New Delhi district with population coverage of 0.1 million. There were 236 and 174 patients with TB (aged ≥20 years) registered for treatment during the study period at North District and New Delhi district DOTS centre respectively. Both DOTS centres had screening of TB and DM operational since January, 2016. Both study sites had clinic for diagnosis and management of NCDs (referred here as NCD clinic).

### Study population

#### Quantitative

All patients with TB aged ≥20 years treated in DOTS centres of two medical colleges between October 2016 and March 2017 in Delhi, were included.

#### Qualitative

All health care providers involved in the screening were interviewed. In addition, we purposively selected patients who underwent screening, aiming at maximum variation in the sample.

### Sample size

Using the yield of a similar screening programme in general population as 6% based on the results of previous studies [18],.minimum sample size was estimated to be 90 with precision of 5% at 95% confidence interval and power of the study as 80%. However, for our study we included every patient coming to DOTS centre for treatment from October 2016-March 2017.

### Study procedures

#### Quantitative

A two stage procedure was followed. After explaining the purpose of study and taking written informed consent, in the first stage, all eligible patients were screened using a questionnaire-based screening tool for NCDs and their risk factors by the DOTS provider. The questionnaire was developed from the WHO STEPS instrument and findings from a previous multi-centre risk factor survey.[19,20] The STEPS instrument is a tool to collect data and measure NCD risk factors within the WHO STEPwise approach to surveillance. The questionnaire was piloted in Ballabgarh block of Haryana, India by Amarchand et al.[18] At the first stage, 9 questions related to age, smoking, alcohol consumption, tobacco (smokeless form), fruit intake, vegetable intake, physical activity, waist circumference and family history of NCDs were assessed. Those who scored more than 8 in first part out of total score of 20, proceeded towards the second stage. In the second stage, measurements of blood pressure, height and weight (for Body Mass Index) were taken using validated procedures [21] by the health worker attached to Department of Community Medicine of the two colleges. Those who scored more than 12 in both stages were referred to NCD clinic for further management. Additionally, the blood (venous) fasting sugar levels of each patient were obtained from the existent records. Operational definitions used for data collection are available in Table 1. Patient who scored less than equal to 12 were given information about prevention and control of NCDs.

**Table 1:** Operational definitions of NCD risk factors and NCDs (DM and hypertension).

Variable	Definition
Tobacco intake[21]	
Current users	Current Smoker/Smokeless Tobacco User was defined as someone who, at the time of the survey, smokes/uses tobacco in any form either daily or occasionally in last 30 days preceding the survey.
Daily users	Current Daily Smoker/Smokeless Tobacco User was defined as someone who smokes/uses tobacco everyday (for last 30 days) with rare exceptions such as days of religious fasting or during acute illness
Past users	Individuals who have had a history of smoking/smokeless tobacco use at any time other than the past 30 days (from beginning of study period).
Ever users	In the present study it means past users and current occasional users
Alcohol intake[22]	
Current Drinker	Current alcohol consumption was defined as one or more than one drink of alcohol consumed in 30 days preceding the survey.
Daily Drinker	Individuals who drink one or more than one drink (men) of alcohol daily (for women more than two drinks)
Past Drinker	Individuals who have had a history of drinking alcohol at any time other than the 30 days (from beginning of study period).
Ever Drinker	In the present study it means past users and current occasional users
Fruit serving[20]	Fruit intake was defined as one or more servings of any seasonal fruit consumed in a day for ≥5 days in a week (serving size 80 grams)
Vegetable serving[20]	Vegetable intake was defined as more than 3 servings of any cooked or raw vegetables respectively consumed in a day.
Physical activity[22]	Adults aged 18–64 should do at least 150 minutes of moderate-intensity aerobic physical activity throughout the week or do at least 75 minutes of vigorous-intensity aerobic physical activity throughout the week or an equivalent combination of moderate- and vigorous-intensity activity.
Hypertension*[23]	Hypertension was defined as blood pressure equal to or greater than 140 mm of Hg systolic and/or 90 mm of Hg diastolic
Diabetes Mellitus[24]	Diabetes mellitus was defined as fasting plasma glucose ≥126 mg% OR 2h Post prandial ≥200 mg% during oral glucose tolerance test OR Hb1ac ≥6.5 OR in patients with classic symptoms of hyperglycemia and random plasma glucose ≥200 mg%
Obesity[25,26]	Overweight and obesity were defined as body mass index (BMI) ≥23–24.9 kg/m^2^ and BMI ≥25 kg/m^2^.Two sets of cut offs were used for defining obesity in terms of waist circumference. These were 90 cm for men and 80 cm for women on the basis of consensus guidelines for obesity and as 102 cm in men and 88 cm in women on the basis of National Cholesterol Education Program Adult Treatment Panel III (NCEP ATP III) recommendations

* For patients ≥ 60 years old, BP>150/90 is taken as hypertension

Note: NCDs: Non-communicable Diseases

#### Quantitative data variables

Data on socio-demographic (age, gender) and clinical characteristics of TB patients [treatment category, type of TB, sputum results at the time of diagnosis, HIV (Human Immunodeficiency Virus) status, fasting blood sugar level] were obtained from their treatment records. Data on presence of NCDs (previous history of NCDs, patients referred to NCD clinic, new cases diagnosed at NCD clinic) and their related risk factors [smoking, alcohol consumption, tobacco (smokeless form), fruit intake, vegetable intake, physical activity, waist circumference and family history of NCDs] were obtained from records of NCD clinic and questionnaire.

#### Qualitative

Perceived relevance and acceptability of the screening programme among TB patients was explored through one to one interview with patients and health providers. The principal investigator (TA) who is trained in qualitative research methods conducted the interviews in Hindi. Participants were informed of the purpose of the study and consent was obtained. Only the interviewee and the researcher were present during the interview. A pre-tested interview guide with broad open ended questions was used. Verbatim notes were taken during the interview. After the interview was over, the summary of the interview was read back to the interviewee to ensure participant validation. The interviews with the patients were conducted immediately after the screening procedure at the DOTS centre. Interviews with health providers were conducted at the health facility in April, 2017.

### Statistical analysis

#### Quantitative

The data were double- entered, validated and analysed using EpiData version 3.1 for entry and version 2.2.2.182 for analysis (EpiData Association, Odense, Denmark). Descriptive analysis was performed in the form of median and inter-quartile range [IQR] (number of risk factors), mean and standard deviation [SD] (for variables age, score of stage 1) or proportions (for categorical variables like socio-demographic, TB related clinical factors, risk factors, patients diagnosed with DM and hypertension etc.) wherever appropriate. Statistical difference between means of score with respect to gender and type of TB, was calculated using the independent t test. The Chi square test/Fischer exact test was used to study association between categorical variables. The associations between the NCDs and risk factors with selected socio-demographic and clinical factors were expressed as odds ratio [unadjusted with 95% confidence intervals (CI)]. Adjusted odds ratios were obtained using log binomial regression in Statistical Package for the Social Sciences (SPSS) version 17.0. P< 0.05 was considered statistically significant. Number needed to screen (NNS = number of persons screened for DM/number of persons newly diagnosed with DM and hypertension) was calculated after excluding already diagnosed cases of DM and hypertension.

#### Qualitative

Interview data were transcribed and translated in English by the PI (TA) on the same day based on the verbatim notes. Transcripts were analyzed using the manual descriptive content analysis by two independent, trained researchers (TA and GK). The decision on coding rules and theme generation was taken by using standard procedures and in consensus of all the investigators. The findings were reported according to Consolidated Criteria for Reporting Qualitative Research.[27]

#### Ethics approval

Ethics approvals were obtained from the Institutional Ethics Committee of the North Delhi Municipal Corporation Medical College, Hindu Rao Hospital, Delhi, India, and the Ethics Advisory Group of the International Union Against Tuberculosis and Lung Disease, Paris, France. For both -the quantitative and qualitative component of the study, written informed consent was taken from patients and health care providers respectively. Confidentiality and privacy of the study participants were assured.

## Results

### Quantitative

#### Socio-demographic and clinical characteristics of participants

Out of the total 410 patients with TB who were eligible to be included in the study, 403 (98%) consented to participate. The mean age (SD) of the patients was 34(13) years. There were 57% male participants. Majority of the participants were newly diagnosed patients (64%) with pulmonary TB (59%). There were 4% (n = 14) patients who were HIV positive. Amongst the participants, 6% (n = 26) and 2% (n = 9) patients were already suffering from diabetes and hypertension respectively (Table 2).

**Table 2:** Socio-demographic and clinical characteristics of TB patients aged ≥20 years treated at 2 DOTS centres in Delhi, India between Oct 2016 –Mar 2017.

Variable	Number (n)	(%)^
Total	403	(100)
**Age**		
20–34 years	257	(64)
35–49 years	84	(21)
≥50 yrs	62	(15)
**Gender**		
Male	231	(57)
Female	172	(43)
**TB treatment category**		
New cases	257	(64)
Retreatment cases	138	(34)
Multi-Drug Resistant cases	8	(2)
**Type of TB**		
Pulmonary	237	(59)
Extra-pulmonary	166	(41)
**HIV status**		
Positive	14	(4)
Negative	389	(96)
**Already diagnosed with**		
Diabetes Mellitus	26	(6)
Hypertension	9	(2)

Note: TB: Tuberculosis; DOTS: Directly observed treatment short course; HIV: Human Immunodeficiency Virus

^ It denotes column percentages

#### NCD risk factors and new diagnosed cases of diabetes mellitus and hypertension

The median (IQR) number of risk factors amongst the study participants was 4 (4–6). Nearly one third of the participants were either smokers (32%) or were using smokeless tobacco (33%) or were consuming alcohol (34%). Only 28% (n = 111) were consuming the recommended amounts of fruit servings in a week while 20% were eating ≥ 3 servings of vegetables in a day. Nearly four-fifths of patients with TB (79%) were physically inactive. There were 107 patients (27%) who were obese. (Table 3)

**Table 3:** NCD risk factors and NCDs detected among TB patients aged ≥20 years treated at 2 DOTS centres in Delhi, India between Oct 2016 –Mar 2017.

NCD Risk factors	n	(%)^
Total	403	(100)
**Smoking status**		
Non smoker	277	(68)
Ever smoker/Smoke occasionally	79	(20)
Daily smoker	47	(12)
**Consumption of smokeless tobacco**		
Non user	271	(67)
Ever user/use occasionally	93	(23)
Daily user	39	(10)
**Consumption of alcohol**		
Non user	264	(66)
Ever user/use occasionally	110	(27)
Daily user	29	(7)
**Fruit consumption**		
≥ 5 days in a week	111	(28)
< 5 days in a week	292	(72)
**Vegetable consumption**		
≥ 3 servings in a day	82	(20)
< 3 servings in a day	321	(80)
**Physical activity**		
Moderate-vigorous PA for ≥10 min/day	83	(21)
Moderate-vigorous PA for < 10 min/day	320	(79)
**Family History of NCDs**		
Yes	92	(23)
No	311	(77)
**Waist circumference**		
Less than 72/78 cm	87	(21)
72-79/78-89 cm	209	(52)
≥ 80/90 cm	107	(27)
**New cases**		
Diabetes Mellitus	6	(2)
Hypertension	20	(5)

NCDs: Non communicable diseases; TB: tuberculosis; DOTS: Directly Observed Treatment, short course; DM: Diabetes Mellitus; PA: Physical Activity

^ It denotes column percentages

**Fig 1:**
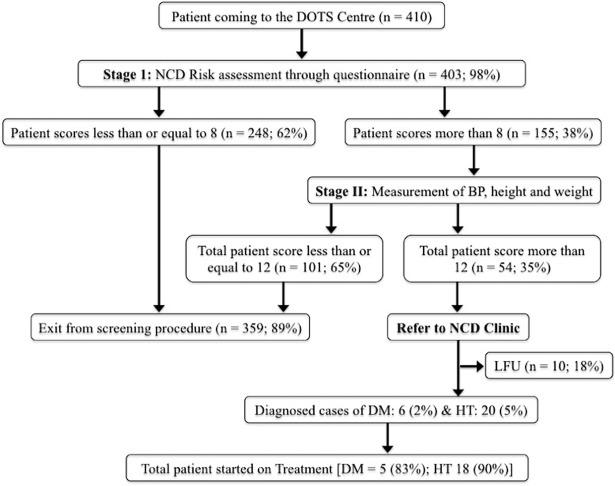
Flow of participants through the screening process.

As shown in Fig 1, of the 403 participants, 38% (n = 155) had a score more than 8 and were enrolled in the stage 2 of the screening. Amongst them, 35% (n = 54) were referred to NCD clinic. Out of the total who were referred, 82% (n = 44) reached the NCD clinic. There were 20 new cases of hypertension diagnosed by NCD clinic while 6 new patients were diagnosed with DM. Out of the newly diagnosed hypertensive and diabetic patients, 90% and 83% were started on treatment, respectively. (Fig 1) The overall prevalence of diabetes and hypertension among the TB patients was 8% and 7% respectively.

The median score (IQR) of stage 1 screening was 8 (6–10). There was significant difference in the stage 1 score with respect to gender (male>female; p<0.001) while it did not differ with type of TB.

The presence of NCDs was found to be associated with age, male sex and pulmonary TB (p<0.05). After adjusting for confounding, increasing age was found to be significantly associated with NCDs. Association was seen between the presence of more than equal to 4 risk factors and age and gender. (Table 4)

**Table 4:** Association between selected socio-demographic & clinical factors and NCDs & risk factors for NCDs amongst TB patients aged ≥20 years, Delhi, India. (Oct 2016 –Mar 2017).

Socio-demographic, clinical variables	Total	NCDs	OR (C.I)	aOR (C.I)	NCD Risk Factors	OR (C.I)	aOR (C.I)
		Present			≥4		
	N	n (%)^			n (%)^		
	403	50			50		
**Age**							
20–34 years	257	12 (5)	1	1	187 (73)	1	1
35–49 years	84	17 (21)	4.6 (2.3–9.1)*	4.2 (1.8–9.7)*	75 (91)	3.1 (1.4–7.1)*	2.9 (1.1–6.5)*
≥50 yrs	62	21 (34)	7.3 (3.8–13.9)*	7.2 (3.2–13)*	59 (95)	7.4 (2.1–30.4)*	7.0 (3.2–19)*
**Gender**							
Male	231	36 (16)	2.1 (1.1–4)*	1.2 (0.6–2.4)	199 (86)	2.5 (1.5–4.2)*	1.9 (1.1–3.3)*
Female	172	14 (8)	1	1	122 (71)	1	1
**Treatment category**							
New cases	257	32 (13)	1	1	200 (78)	1	1
Re-treatment cases	138	17 (12)	1.1 (0.6–1.8)	1.0 (0.2–4.5)	116 (84)	1.5 (0.8–2.7)	0.6 (0.2–1.7)
MDR TB cases	8	1 (13)	1.0 (0.2–6.5)	1.0 (0.4–2.6)	5 (62)	0.5 (0.1–2.6)	0.5 (0.3–1.1)
**Type of TB**							
Pulmonary	237	38 (16)	2.5 (1.2–4.8)*	1.7 (0.8–3.6)	128 (77)	1.3 (0.8–2.1)	1.0 (0.6–1.6)
Extra-pulmonary	166	12 (7)	1	1	193 (81)	1	1
**HIV status**							
Positive	13	1 (7)	1.9 (0.2–14.6)	1.2 (0.2–10.2)	12 (86)	0.6 (0.1–2.9)	0.6 (0.1–2.9)
Negative	340	49 (13)	1	1	309 (79)	1	1

*p<0.05

NCDs: Non communicable diseases; TB: tuberculosis; OR: Odds Ratio; aOR: Adjusted Odds Ratio; C.I: Confidence Interval; MDR TB: Multi-Drug Resistant Tuberculosis; HIV: Human Immunodeficiency Virus

^It denotes row percentages

#### Number needed to screen for diabetes mellitus and hypertension

The NNS for diabetes was 63 while 20 TB patients needed to be screened to get one new case of hypertension. Risk factor wise stratification revealed that NNS was less if the study population comprised of males, smokers, consumers of smokeless tobacco, alcoholics and obese for both the diseases. (Table 5) The NNS amongst patients (n = 155) who underwent stage 2 screening was 68 and 7 for diabetes and hypertension respectively.

**Table 5:** Number needed to screen (NNS) for Diabetes mellitus and hypertension according to some selected risk factors.

Variables	Diabetes Mellitus	Hypertension
**Total participants**	377	394
**Overall**	63	20
**Age**		
20–34 years	51	51
35–49 years	NA	14
≥50 years	48	11
**Gender**		
Male	35	14
Female	NA	41
**Smoking status**		
Non smoker	44	30
Smoker	NA	11
**Consumption of smokeless tobacco**		
Non user	85	29
User	41	12
**Consumption of alcohol**		
Non user	84	64
User	41	9
**Waist Circumference**		
Less than 79/89 cm	93	87
≥ 80/90 cm	33	16

NA: Not applicable

### Qualitative

**Fig 2:**
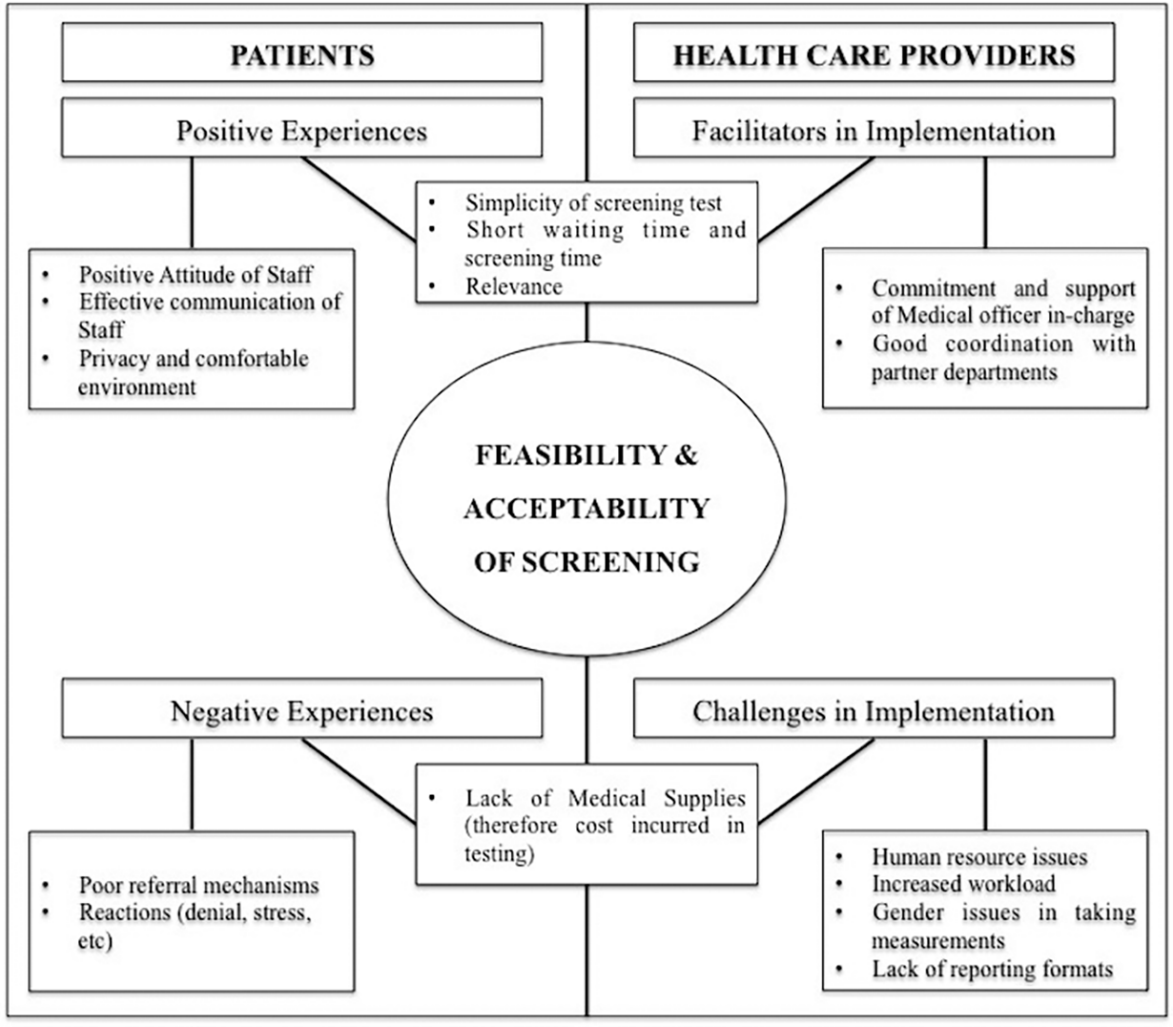
Percieved acceptability and relevance of screening of NCDs and their risk factors amongs TB patients and health providers.

A total of 20 one to one interviews were conducted (16 with patients and 4 with health providers). While the participants shared their experiences of undergoing an integrated screening process, health providers discussed various enablers and challenges in implementation of screening. The average duration of the interviews was 18 minutes (15–30 minutes) From the transcripts, 19 codes were identified and they were grouped into four main themes: 1) positive and 2) negative experiences of patients, 3) facilitating factors and 4) challenges in implementation as perceived by health providers. We illustrated in a non-hierarchical way, the themes and associated codes in Fig 2.

#### Positive experiences of patients

Almost all participants considered that the screening process was simple and smooth. The waiting time and time to undergo screening varied from 5–10 minutes. Patients remarked that the questions asked to them were easily understandable and considered screening for NCDs very relevant. One patient remarked “*we are already coming to the health facility and if we are checked for any other disease simultaneously*, *it is very useful*. . . .*diseases can be detected early*.” Another interviewee said; “*As such after getting TB*, *I think I should undergo regular check-up*. . . ..*and if we are getting it free here*, *what is the harm*?.”. The positive attitude of staff involved and their effective communication skills were reported as important elements of the screening process. Except for one participant, the interviewees found the screening room to be comfortable and their privacy assured.

#### Negative experiences of patients

There were several undermining factors that led to some negative experiences by patients. Waiting time and out of pocket expenditure for the blood glucose testing emerged as major issues. The cost of test at private laboratories varied from Rs.10 to Rs.50. *“It is not possible to go here (hospital) as there are long queues and I don’t have time*. . . ..*”*, one participant remarked. Some participants who were referred to NCD clinic cited long waiting time at the hospital as a deterrent to their visit. Additionally, they had to have an OPD (out-patient department) slip which meant standing in another queue.

The general reaction of the participants to disclosure of high risk status was that of distress and denial. The extent of distress was expressed by one of the participant as *“I do not know what to do now*. *I got TB and diabetes at young age and now high BP*. . . ..*it feels terrible*. . . ..*now I will have to take another pill*.*”* Another remarked *“my sugar levels have always come normal*. *How can it be high now*?. . . . . .*I do not agree I have diabetes*. *I think lab report is wrong”*. The need for counselling and patient support emerged as an essential component of such a screening procedure despite the non-stigmatizing nature of such diseases.

#### Facilitators in implementation according to health providers

Similarly to patients, health providers, found the screening tool to be simple and short. Short waiting and screening time was reported from both participating health facilities. Health providers considered integrated screening of NCDs relevant in the present context. One of them pointed out *“when DM-TB screening was started*, *we were told in our training that TB patients have higher risk of getting diabetes* … *Similarly*, *by measuring BP we may find that TB patients may be at risk of high BP as well* …*Even if not*, *such screening will help in earlier detection of disease*.*”* The strong support, coordination and commitment they received from their respective departments as well as partner departments were highly valued as enabling factors by the interviewees.

#### Challenges in implementation according to health providers

Participants had mixed feelings regarding perceived workload after the implementation of the integrated screening. *“*..*with daily regime and DOTS99 [it is a scheme using mobile phones to monitor adherence to TB medications] coming soon*, *my work is going to increase*. *TB related work is too much* …*thinking of implementing this would add to my already exhaustive work*. . . .*An additional person can be designated for this*.*”*, one of them remarked. Health providers also expressed their concern regarding inadequacy of knowledge and skills they possess.

One of the health providers brought a particular gender issue related to anthropometric measurements; *“Since I am male*, *I could not take [waist circumference] measurements of females*. *So I had to ask my colleague to do the same*. . . .*But this is not possible every time*. *A female health worker is required in examination of female patients*.*”*

Medical logistics and a well-functioning supply chain are essential elements for service provision. Non availability of glucometers at both the DOTS centre was reported, because of which the patients had to get their blood sugar testing done from outside. One health provider remarked *“I do not think my centre is fully equipped with medical supplies like BP apparatus*, *glucometer and measuring tape*. *For TB-DM screening also*, *I had to ask patients to get their sugars done from outside*. *Some of them had to pay for such tests*.*”* This last point emerged as the most negative experience from the patients’ perspectives as well. Health providers also pointed out that unlike reporting of TB in the programme, there is lack of standardized reporting formats for DM services in the programme.

The interviewees provided several suggestions and recommendations for the improvement of the current screening process as well as for the implementation of future intervention. These recommendations are enlisted in Table 6.

**Table 6:** Suggestions and recommendations by health providers for implementation of integrated screening programme.

Challenge	Solutions
Overburdening of DOTS provider; lack of medical supplies; Gender issues	• The screening can be implemented at the level of chest clinic where the staff is more. • Further, the screening can be standardized to be organized at the point of diagnosis. • Gender related issues can also be resolved at that level
Lack of knowledge and skills about integrated screening among the health provider	• Training and re-training of the staff • Involvement of health providers who have good experience in provision of integrated service
Lack of reporting formats	• Incorporation of important parameters of screening in existent HMIS

DOTS: Directly Observed Treatment, short course; HMIS: Health Management Information System

## Discussion

This study provides supportive quantitative and qualitative evidence on the feasibility and acceptability of integrating screening for NCDs and NCD risk factors within routine TB care at the level of DOTS centre. This is particularly encouraging in the present context, with a large, dual burden of TB and NCDs in India.

The overall prevalence of diabetes in this TB cohort in Delhi was 8%, of whom 2% were newly diagnosed cases. This is much lower than reported by several studies from the Southern India.[28,29] However, findings by India Tuberculosis-Diabetes Study Group (ITDSG) in North India and from a similar study in a tertiary care hospital in Bangalore showed comparable prevalence.[30,31] The lower prevalence of DM among TB patients reported in our study could be due to lower mean age of our population than in other studies and partly due to the heterogeneity in prevalence of diabetes across geographic areas and ethnic groups. The NNS to detect a new case of DM in our study was 63. This is much higher than reported by studies conducted in Kolar and Bangalore.[11,30] This could be attributed to already implemented universal screening strategy for TB-DM.

While the prevalence of DM among TB patients is well documented, there is very limited evidence available for prevalence of hypertension among TB patients in India.[32] The prevalence of hypertension in the present study was 7%. According to the existing literature on the occurrence of hypertension amongst TB patients across the world the prevalence varies from 0% to 50%.[33] Therefore, more evidence is required to draw valid conclusions. In any case, the yield of screening for hypertension in our patients was high and this presents a strong case for integration of NCDs and TB programme as evidenced in other parts of the country as well.[32]

Our study also revealed high prevalence of NCD risk factors like smoking, and consumption of smokeless tobacco and alcohol. The findings corroborate with studies done elsewhere in similar populations.[32,34,35] Smoking is an important risk factor for development of TB. This confluence act as catalyst for development of NCDs in such patients. Therefore, such high prevalence of smoking in this population implies impending burden of NCDs amongst them. Low consumption of fruits and vegetables by our study population is worrisome, particularly when compared to general population.[36] The inadequate intake of fruits and vegetables not only pose as risk factors for NCDs, but it is an important determinant of clinical outcomes of TB.[37,38] Therefore, the finding needs to be addressed for optimal management of TB. Lastly, the majority of our study population was physically inactive. This is in line with the evidence that suggests TB affects the physical functioning along with many other dimensions of life due to physical de-conditioning, muscle atrophy, and impaired lung function and gas exchange.[39,40] More than a quarter of TB patients in our study had central obesity which points towards increased risk for diabetes.[41]

As expected, age and male sex were found to be significantly associated with the presence of NCDs and their risk factors in the current study. This goes in line with the existing evidence.[41] Prevalence of TB is more in males as compared to females. So, it very likely, that more males were found in our study. Further, epidemiology of TB suggests, it mostly affects young people who are in economically productive age group. It may affect some aspects such as certain risk factors such as tobacco use and alcohol intake might be more common among males. However, to avoid bias due these, an adjusted analysis has also been undertaken.

One of the main stakeholders in successful implementation of any screening are the users; and it is essential that their perspectives and experiences are assessed and analysed. The patients in our study considered the screening relevant and supported the procedures without any resistance. Evaluation of TB-DM screening across India endorse the same finding.[42] However, lack of medical supplies and cost incurred on blood tests were justifiably bothersome for some patients. This has been considered as one of the challenges by the health providers as well. Integrated health service requires a continuous supply of medical equipment, reagents and drugs.[13]

While the patients in our study opined that referral mechanisms were time consuming, health providers perceived them to be smooth. These conflicting opinions are explained by the different roles the two parts play in the health system. And it is important to understand patients’ perspectives and experiences while planning for any health services for them. It also provides insights into the extent to which the patient expectations of care have been attained.[43]

The overall positive attitude observed amongst health providers towards provision of integrating NCDs with TB indicates their acceptance for such services in the future. However, they acknowledged increased workload and shortage of skilled and experienced staff as the main challenges they faced while implementing the screening. Providers also stated the absence of structured and standardized reporting formats for NCD services as previously reported from pilot studies in India and China.[31,44]

Several suggestions were given by health providers. Firstly, the screening for NCDs could be done at the level of tuberculosis unit or chest clinic. This will help to make the screening more standardized at the point of diagnosis. There is sufficient evidence regarding screening TB patients for DM earlier in the course of TB treatment.[31] Further, it will also help to address the shortage of staff and sharing of workload or task shifting is also possible at that level. Secondly, training and re-training of staff would aid in enhancing the quality of services provided. Lastly, they suggested to include important parameters for NCDs and NCD risk factors in existent TB reporting formats. This would enable better monitoring of such services.

The study had several strengths. Firstly, we employed a mixed methods design which gave a comprehensive view about the feasibility, relevance and acceptability, both in quantitative and qualitative terms. Secondly, the study used validated tools and methods for collection of data and standardized operational definitions. Thirdly, the screening was implemented within the routine system with no additional budget and this adds to the pragmatic nature of the study. Lastly, since the data were collected prospectively, there were minimal missing data. However, there were some limitations as well. The study was conducted in two DOTS centre attached to medical colleges in Delhi. The level of training, knowledge amongst providers and type of patients may be very different from other populations. Therefore the findings may have limited external validity. Secondly, absence of control or comparison group leads to selection bias. This can further limit the validity of findings. Thirdly, the qualitative data was transcribed from verbatim notes and translated from Hindi to English. This could limit the validity and transferability of study findings. Further, we interviewed only health providers at the level of DOTS centre. Interview with other stakeholders like programme managers who are actually involved in programme implementation could have given different perspectives.

Limitations notwithstanding, the study provided useful insights into integrating NCDs in routine TB care. This has the potential to lead to efficiency in health care delivery and benefits to the patients. The existing well-functioning RNTCP and readiness and relevance of such screening as opined by health providers and patients offer a good opportunity for integrating NCD screening with TB.

## Supporting information

S1 File Guidelines for taking measurements and questionnaire. (DOCX)
