# Supplementary material for: Integrating screening for non-communicable diseases and their risk factors in routine tuberculosis care in Delhi, India: A mixed-methods study
Source: PLoS One. 2018 Aug 23;13(8):e0202256. doi: 10.1371/journal.pone.0202256 (PMC6107155; doi:10.1371/journal.pone.0202256)
Supplement: S1 File — (DOCX) [file pone.0202256.s001.docx]

**SUPPLEMENTAL MATERIAL**

**Guidelines for taking measurements using validated methods** (25)

Where possible, all physical measurements should be conducted in a private area.

1. **Waist circumference:** This measurement should be taken without clothing, that is, directly over the skin. If this is not possible, the measurement may be taken over light clothing. Standing to the side of the participant, locate the last palpable rib and the top of the hip bone. Ask the participant to wrap the tension tape around themselves and then position the tape at the midpoint of the last palpable rib and the top of the hip bone, making sure to wrap the tape over the same spot on the opposite side. Ask the participant to: stand with their feet together with weight evenly distributed across both feet;hold the arms in a relaxed position at the sides; breathe normally for a few breaths, then make a normal expiration. Measure waist circumference and read the measurement at the level of the tape to the nearest 0.1 cm, making sure to keep the measuring tape snug but not tight enough to cause compression of the skin. Record the measurement
2. **Height:** Ask the participant to remove their: footwear (shoes, slippers, sandals, etc), head gear (hat, cap, hair bows, comb, ribbons, etc). Ask the participant to stand with: feet together, heels against the back board, knees straight, look straight ahead and not tilt their head up. Make sure eyes are the same level as the ears. Move the measure arm gently down onto the head of the participant and ask the participant to breathe in and stand tall. Record the height measurement in centimeters.
3. **Weight:** Ask the participant to remove their footwear (shoes, slippers, sandals, etc) and socks and step onto weighing scale with one foot on each side of the scale. Ask the participant to: stand still, face forward, place arms on the side and wait until asked to step off. Record the weight in kilograms.
4. **Blood pressure:** Ask the participant to sit quietly and rest for 15 minutes with his/her legs uncrossed. Three blood pressure measurements should be taken. During data analysis the mean of the second and third readings will be calculated. The participant will rest for three minutes between each of the readings. Place the left arm of the participant on the table with the palm facing upward. Remove or roll up clothing on the arm. Select the appropriate cuff size for the participant. Position the cuff above the elbow aligning the mark ART on the cuff with the brachial artery. Wrap the cuff snugly onto the arm and securely fasten with the Velcro. The lower edge of the cuff should be placed 1.2 to 2.5 cm above the inner side of the elbow joint. Keep the level of the cuff at the same level as the heart during measurement. Switch the monitor on (dark purple button) and press START (light purple button). The monitor will start measuring when it detects the pulse and the "heart" symbol will begin to flash. The systolic and diastolic blood pressure readings should be displayed within a few moments (systolic above and diastolic below). Record the reading in the participant's instrument. Switch the monitor off, but leave the cuff in place. Wait three minutes, then repeat steps 1-4 two more times.

**Annexure 2: Individual Non-communicable Disease (NCD) Risk Determinant Questionnaire**

I. Personal Information

1. TB Reg. No: __________________________________
2. Age (in completed years): __________________
3. Gender: Male/Female

II. Treatment History

1. Category of treatment: ___________________________
2. Date of start of treatment: _______________________
3. Phase of treatment: _______________________________
4. Result of sputum at the start of treatment: ___________________________

Record any other observation: _______________________________________________

III. Have you ever been diagnosed with any of the following disease?

| Hypertension | Heart Disease | Diabetes | Stroke | Chronic Respiratory Disease | Cancer |
| --- | --- | --- | --- | --- | --- |
| Yes/No | Yes/No | Yes/No | Yes/No | Yes/No | Yes/No |

If any of the above answer is yes, kindly guide the patient about the treatment and appropriate treatment facility

IV. NCD Risk Determinant Questionnaire-Stage I

| Question | Options | Score | Record the score |
| --- | --- | --- | --- |
| 1. What is your age (in completed years)? | 20-34 years | 0 |  |
|  | 35-49 years | 2 |  |
|  | More than equal to 50 years | 4 |  |
| 2. Do you smoke?  If yes, do you smoke daily?  If no, have you ever smoked? | Never | 0 |  |
|  | Ever smoked or sometimes smoke | 1 |  |
|  | Daily | 2 |  |
| 3. Have you ever taken tobacco such as Gutkha, Khaini etc.?  If yes, do you use tobacco daily?  If no, have you ever used tobacco? | Never | 0 |  |
|  | Ever taken or take sometimes | 1 |  |
|  | Daily | 2 |  |
| 4. Have you ever taken alcohol?  If yes, do you take alcohol daily?  If no, have you ever taken alcohol? | Never | 0 |  |
|  | Ever taken or take sometimes | 1 |  |
|  | Daily | 2 |  |
| 5. How many days in a week do you consume fruits? | More than equal to 5 days | 0 |  |
|  | Less than 5 days | 1 |  |
| 6. How many servings of vegetable do you consume in a day? | More than equal to 3 | 0 |  |
|  | Less than 3 | 1 |  |
| 7.a Do you daily do moderate (in which your only your heart beat fastens like climbing stairs, cycling, brisk walking) or  b. vigorous (in which your heart beat fastens and you become breathless | Moderate to vigorous physical activity for more than equal to 10 min | 0 |  |
|  | Moderate to vigorous physical activity for less than 10 min | 2 |  |
| 8. Do any of your parents or brother or sister has history of high blood pressure, diabetes, or heart disease? | No | 0 |  |
|  | Yes | 2 |  |
| 9.Waist circumference (in cm): ________________________________ | Less than 72/78 | 0 |  |
|  | 72-79/78-89 | 2 |  |
|  | More than equal to 80/90 | 4 |  |

Note: If the total score is more than 8, then move to next part

V. Measurements: Stage II

| Measurements | Options | Score | Record the score |
| --- | --- | --- | --- |
| 1. Blood Pressure   1^st^ Reading: _____________  2^nd^ Reading: _____________  3^rd^ Reading: _____________ | Less than 140/90 | 0 |  |
|  | 140-160/90-100 | 2 |  |
|  | More than equal to 160/100 | 4 |  |
| 1. Body Mass Index   Height (in cm): ____________  Weight (in Kg): ____________ | Less than 23 | 0 |  |
|  | 23.0-24.9 | 2 |  |
|  | More than equal to 25 | 4 |  |

Note: If the total score is more than to 12 refer the patient to NCD clinic

Record the Blood Sugar Finding here:

1. Fasting Blood Glucose (in mg/dl): _____________________________
